# Comparison of Sample Preparation Methods Used for the Next-Generation Sequencing of *Mycobacterium tuberculosis*

**DOI:** 10.1371/journal.pone.0148676

**Published:** 2016-02-05

**Authors:** Andrea D. Tyler, Sara Christianson, Natalie C. Knox, Philip Mabon, Joyce Wolfe, Gary Van Domselaar, Morag R. Graham, Meenu K. Sharma

**Affiliations:** 1 National Microbiology Laboratory, National Reference Centre for Mycobacteriology, Public Health Agency of Canada, Winnipeg, Manitoba, Canada; 2 Science Technology Cores & Services Division, National Microbiology Laboratory, Public Health Agency of Canada, Winnipeg, Manitoba, Canada; 3 Department of Medical Microbiology, University of Manitoba, Winnipeg, Manitoba, Canada; 4 Department of Computer Science, University of Manitoba, Winnipeg, Manitoba, Canada; Institut Pasteur de Lille, FRANCE

## Abstract

The advent and widespread application of next-generation sequencing (NGS) technologies to the study of microbial genomes has led to a substantial increase in the number of studies in which whole genome sequencing (WGS) is applied to the analysis of microbial genomic epidemiology. However, microorganisms such as *Mycobacterium tuberculosis* (MTB) present unique problems for sequencing and downstream analysis based on their unique physiology and the composition of their genomes. In this study, we compare the quality of sequence data generated using the Nextera and TruSeq isolate preparation kits for library construction prior to Illumina sequencing-by-synthesis. Our results confirm that MTB NGS data quality is highly dependent on the purity of the DNA sample submitted for sequencing and its guanine-cytosine content (or GC-content). Our data additionally demonstrate that the choice of library preparation method plays an important role in mitigating downstream sequencing quality issues. Importantly for MTB, the Illumina TruSeq library preparation kit produces more uniform data quality than the Nextera XT method, regardless of the quality of the input DNA. Furthermore, specific genomic sequence motifs are commonly missed by the Nextera XT method, as are regions of especially high GC-content relative to the rest of the MTB genome. As coverage bias is highly undesirable, this study illustrates the importance of appropriate protocol selection when performing NGS studies in order to ensure that sound inferences can be made regarding mycobacterial genomes.

## Introduction

Application of next-generation sequencing (NGS) to the study of clonal, slowly evolving microorganisms such as *Mycobacterium tuberculosis* (MTB) via whole genome sequencing (WGS), has led to improvements in epidemiological tracking of outbreaks, and aids in clarifying transmission patterns that cannot be confidently resolved using conventional locus-based genotyping methods. Indeed, the decreasing cost and increasing accuracy, efficiency, resolution, and reproducibility of NGS technologies have made large-scale WGS of target organisms, not only feasible for basic research, but also applicable for surveillance and response activities. However, to maximize the utility of this technology, consideration of the possible biases and limitations of the experimental methodology employed is essential. Furthermore, knowledge of potential sequencing biases is necessary to ensure that appropriate downstream analytical tools, models, experimental variables and statistical methods are appropriately utilized.

Analyses of clonal isolates require that even minor variability between highly genetically-similar isolates is detectable in order to fully resolve chains of transmission. The current standard of practice for WGS studies investigating the relationship between a set of isolates, involves identifying single nucleotide variants (SNV), compared to a reference sequence [1], and using the cumulative data from these SNV loci to infer the phylogenetic distance and evolutionary relationship between isolates [2]. In cases of slowly diverging organisms that form monomorphic populations, such as MTB, the ability to capture all available genomic diversity is crucial. Accurate identification of true variants versus variants owing to sequencing error requires relatively uniform high-level depth of sequencing (read) coverage across the genome for each isolate included in the analysis. Variability in the read coverage depth across a genome may decrease the amount of information available for global analysis, and limits the true variability detectable in a sequencing experiment. To this end, selection of appropriate experimental protocols to generate robust, high quality data and thus maximize sequence data usability is essential.

Studies using Illumina sequencing-by-synthesis (SBS) technology dominate the field of bacterial WGS[3][4][5]. In the case of microbial WGS, the majority of studies use Illumina MiSeq technology employing either Nextera XT (NX) or TruSeq (TS) Sample Preparation Kits (Illumina, San Diego, USA) for library construction prior to sequencing. Each of these relies on construction of NGS libraries, but by different mechanisms. The NX kit fragments genomic DNA (gDNA) employing a proprietary transposon/transposase-mediated cleavage mechanism, with genomic fragments subsequently amplified using primers targeted to adaptor sequences linked to the transposon. In contrast, in the TS protocol gDNA is first fragmented by mechanical shearing, followed by end-repair of the fragments and adaptor ligation. Advantages to using the NX kit include the requirement for only 1ng of input DNA and significantly faster preparation time [6]. Although several studies have described certain genomic traits that are especially difficult to sequence, such as regions of extremely high or low GC content [7][8], specifically associating with the GGCxC motif [9], none have attempted to fully quantify additional organism- and experiment-specific factors that may be important in WGS analyses. Furthermore, the effect of DNA template quality and both DNA extraction and library preparation methods on sequencing bias has yet to be well described.

To address these issues, we performed a WGS experiment in the high GC-content organism *Mycobacterium tuberculosis* (MTB), and evaluated the effect of library preparation method on Illumina MiSeq sequence data quality. The goal of this study was to ascertain which experimental and/or microbe-specific factors might influence WGS data quality. To investigate this, the NX and TS genomic library preparation protocols were tested in parallel to determine whether either provided more uniform, high quality, sequencing depth of coverage for MTB isolates. Additionally, the effect of DNA purity on sequencing depth of coverage was evaluated. Finally, we investigated the composition of the bacterial genome in regions of high and low read coverage to determine whether sequence-specific factors may contribute to data quality.

## Materials and Methods

Samples included in this analysis were part of a larger WGS study of Canadian MTB isolates, which were obtained by the National Reference Centre for Mycobacteriology (Winnipeg, MB, Canada) for molecular surveillance. A total of 72 isolates collected from 2003 to 2014 were randomly selected for inclusion in this analysis. Selected strains were cultured on Lowenstein-Jensen slants (in-house) using standard, aerobic growth conditions. Cultured MTB was harvested, and DNA was extracted using the MasterPure Complete DNA & RNA Purification kit by Epicentre (Mandel Scientific, Guelph, Canada), which includes Proteinase K treatment of cell suspensions. Additionally, a ten minute boiling step was applied to ensure all samples were no longer viable for bacterial growth. DNA was quantified using PicoGreen (Life Technologies, Burlington, Canada) or Qubit (Life Technologies, Burlington, Canada).

Manufacturer suggested protocols for generation of TS and NX libraries were followed, and reactions included the recommended amount and concentration of DNA (1ng for NX, and 200ng for TS)[10,11]. The MTB samples were multiplexed using Illumina-supplied barcodes, and DNA pools were size-selected to be in the range of 600–1000 bp (average peaks of ~800 bp) using the BluePippin (Sage Science, Beverly, USA). Paired-end sequencing was performed on the Illumina MiSeq. TS samples were only sequenced using the 600-cycle (MiSeq Reagent Kit v3) sequencing kit format (*n* = 72). For NX-based libraries, both 500 (NX-500. MiSeq Reagent Kit v2) (*n* = 25) and 600 (NX-600. MiSeq Reagent Kit v3)-cycle (*n* = 47) sequencing kits were tested. Based on the Lander/Waterman equation for estimating per base coverage of a standard MiSeq run using the 600-cycle kit, with 24 samples multiplexed, we estimated having the potential to generate 145x average coverage across the 4.3-million base pairs (Mbp) of the MTB genome. For the 500-cycle kit, in which 16 samples were multiplexed, we estimated having 73x coverage. Fastq reads generated in this study have been submitted to the NCBI Sequence read archive (SRA) under Bioproject SRP064127 (PRNJA295328).

### Evaluation of sequencing depth of coverage across the MTB genome

Following sequencing, Galaxy was used to calculate average per base PHRED quality scores for forward and reverse reads using FastQC (version 1.2)[12]. Paired-end reads were merged using FLASH (version 1.3.0)[13] followed by d*e novo* assembly using SPAdes v1.0 [14] and annotation using PROKKA v1.4.0([15]). Reference-based assembly was conducted using SMALT (version 0.0.3), with word length and step size equal to 13[16] and H37Rv (NC_018143.2) used as the reference genome. Under default SMALT parameters, sequences mapping equally well to multiple genomic regions were randomly assigned to a location during pileup construction.

Contigs with less than an average depth of coverage of 30x were removed from the *de novo* assembly, and regions meeting a sufficient depth of coverage were visualized via creation of a pangenome with GView server [17]. Per base depth of sequencing coverage for unfiltered BAM files from the reference-based assembly, was calculated using GATK and BEDtools [18], with genomic location established using the H37Rv (NC_018143.2) MTB reference strain as a genome map. Basic statistics including mean, median, and quartile range values were calculated. Per base depth of coverage data was used to construct a non-metric multidimensional scaling (MDS) plot in which the location of points in multidimensional space was determined based on the Euclidian distances between samples, calculated from the sequencing coverage depth at each position across the genome, using the MASS package in R. Differences between coverage in the NX and TS samples were compared using the Wilcoxon signed-rank and the exact McNemar tests of significance.

### Sample quality in relation to data quality

In order to determine whether sequence quality was influenced by DNA sample quality, sample purity was assessed spectrophotometrically using the Nanodrop (Thermo Scientific, Willmington, USA), and correlation with mean sequencing coverage depth in both NX and TS, was assessed. In a subset of samples in which WGS data was available, the ratio of absorbance at 260 and 280 nm (A_260/280_) values were used as a proxy to measure proteinaceous contaminants, and the ratio of absorbance at 260 and 230 nm (A_260/230_) to measure EDTA, organics and other carbohydrates [19]. Pearson correlation was used to evaluate the relationship between variables. Additionally, two samples were extracted and split for pairwise treatment: one pool was treated to an additional column-based wash step (NucleoSpin gDNA Clean-up XS, Macherey-Nagel, Toronto, Canada); the remaining pool maintained as per the standard protocol (did not undergo additional processing). Samples then underwent library preparation using NX or TS protocols, and 600-cycle MiSeq sequencing, and were compared for sequencing quality.

### Identifying genomic regions of biased sequencing depth of coverage

In order to determine whether specific genomic features were associated with reduced depth of coverage, we attempted to identify whether there were common genomic regions in which low coverage was observed across multiple MTB isolates. In a cohort of equal numbers of NX (combined) and TS samples (*n* = 48), which were selected based on the order in which sample sequencing occurred, sequence regions of “ultra-low” coverage (ULC) were identified, defined as regions of at least 10-bp in length in which less than 5x sequencing depth of coverage were achieved across each of the bases in the segment. All ULC regions that were present in ≥5 MTB isolates were then evaluated to see how commonly they occurred across isolates sequenced as NX or TS libraries, using the McNemar paired test of significance. Regions that were <10 bp long, and present in less than 5 MTB isolates were documented as regions of sporadic low depth of coverage (SLC) but were not included in statistical analyses. DiagnoseTargets (GATK) was used to determine the GC-content of the identified genomic regions of ULC depth. Sequence motifs that were more common in regions of ultra-low depth of coverage were identified using GLAM2 in MEME (4.10.0)[20]; AME (4.10.0)[21] was used to determine whether these motifs occurred significantly more frequently in regions of ULC depth than in regions of higher depth of coverage (>20x coverage in both the NX and TS platforms), using a Fisher’s exact test with conservative Bonferroni correction for multiple testing. Finally, all regions commonly observed to show poor coverage across isolates were annotated using bedops v2.4.11[22].

Previous work by Adey et al [23] had demonstrated a slight bias in the native *Tn*5 transposase activity (used in NX library protocol) toward a base signature comprised of AGNTYWRANCT. To determine whether this motif or that resulting from our motif analysis, were more or less frequently detected in MTB compared to other organisms, we counted the number of occurrences of each genetic signature sequence across the forward and reverse strands of completed genomes for several species using a custom Perl script. *Escherichia coli* K-12 (NC_000913.3), *Pseudomonas fluorescens* UK4 (CP008896.1), *Staphylococcus aureus* NCTC8325 (NC_007795.1), *Corynebacterium diphtheriae* HC04 (NC_016788.1), and *Mycobacterium canettii* CIPT (NC_015848.1) were selected for this analysis based on their varying GC-content (range 32–65% GC) and phylogenetic distances to MTB. The frequency of occurrence of the patterned base signatures in each genome was then normalized based on the total size of each bacterial genome under study.

All statistical analyses were performed using R (version 3.0.2).

## Results

### Comparison of Sequence Quality

As expected, the average PHRED quality scores (10-base increments) across the forward and reverse reads decreased towards the end of the Illumina reads, with a more dramatic reduction in quality observed in the reverse read. However, this trend was much less apparent among the TS samples relative to the NX data sets. Furthermore, the end-read sequence quality of the NX-600 samples was much higher than that of the NX-500, approaching that seen in the TS data (S1 Fig).

**Fig 1:**
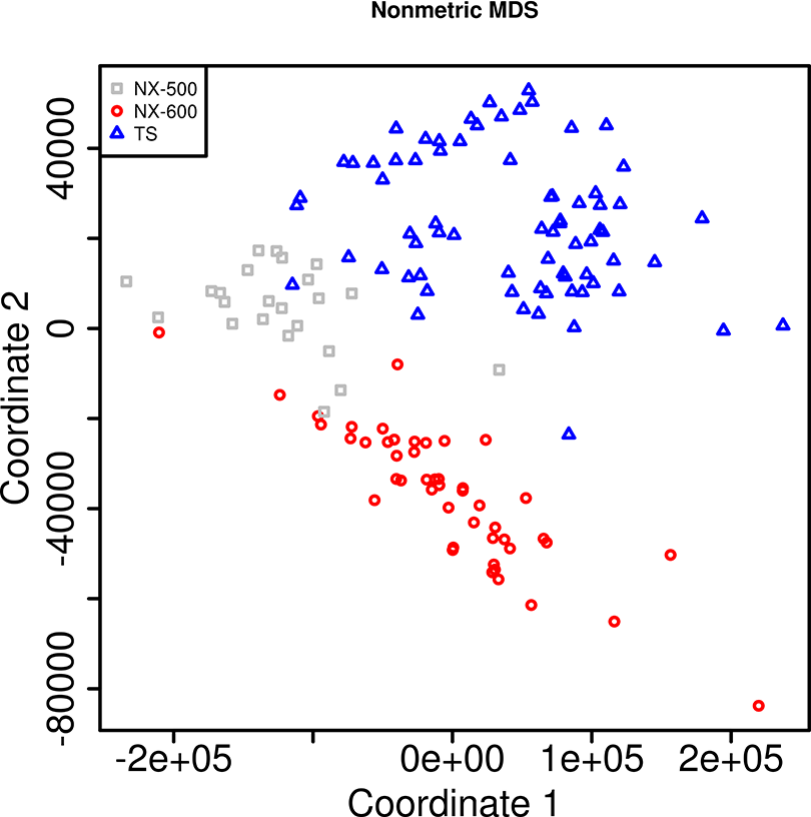
Depth of Coverage, Non-metric Mulitdimensional Scaling plot. Plot illustrates the Euclidian distance between isolates, based on the sequencing depth of coverage obtained for each isolate at each position across the reference H37Rv MTB genome (NC_018143.2). Each sample is colored by the sample preparation method used.

**Fig 2:**
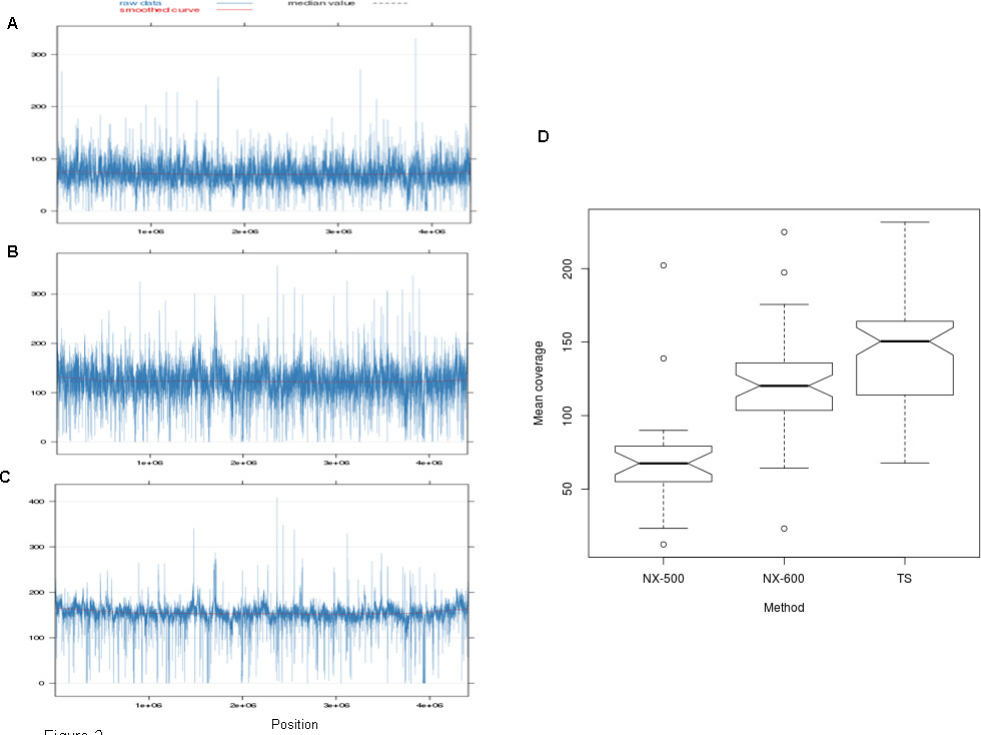
Evaluation of the sequencing depth of coverage across the three library preparation methods explored in this experiment. Coverage across H37Rv MTB reference genome (NC_018143.2) using A) the NX-500 method B) the NX-600 method C) the TS method (600 cycle). D) Illustrates the mean values across the genome via each of the methods. Isolates prepared using TS had significantly higher depth of coverage across the genome than both NX-500 and NX-600 (*p*<0.05).

**Fig 3:**
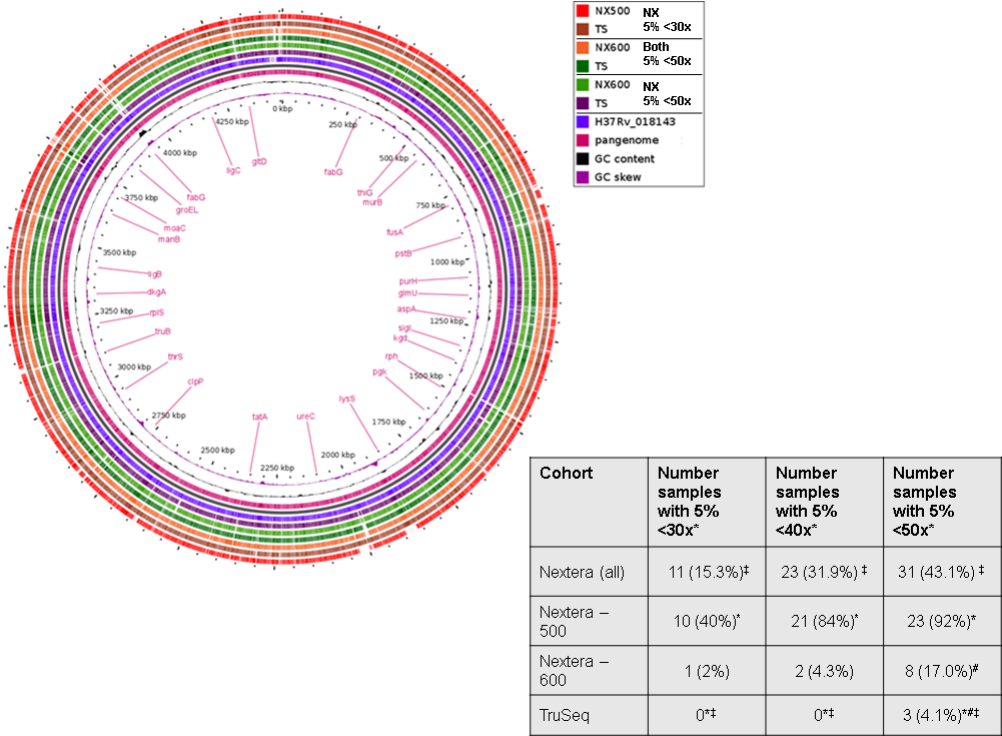
A) Pangenome analysis of the assembled whole genome sequence of two MTB isolates sequenced for this analysis. A cutoff average of 30x depth of coverage was used for inclusion of genomic regions in this image. B) Comparison of the number of isolates in which less than 5% of the genome fails to meet the specified depth of coverage. Symbols illustrate the observation of significant differences in proportion of samples between sequencing methods calculated using the paired McNemar test (p<0.05).

Clustering with multidimensional scaling (MDS) showed distinct grouping of read sets by library preparation method, suggesting that there were large-scale differences in the sequencing depth of coverage across the isolates that were sequenced using NX-500/ NX-600 and TS (Fig 1). When we further explored the depth of coverage across the genome, more uniform coverage depth was observed for the TS samples (Fig 2; S1 Table). The average sequencing read depth of coverage was relatively high across all methods at 71.2 (SD 36.4), 120.6 (SD 32.5) and 142.1 (SD 34) for NX-500, NX-600 and TS respectively (SD values represent standard deviation of the reported mean). These values were all below the theoretical expected coverage for both the 500 and 600 cycle MiSeq reactions previously mentioned (namely 73x and 145x respectively). Samples prepared using TS had significantly higher mean read coverage than did those obtained using both NX-500 (*p*_wilcoxon_ = 1.2x10^-4^) and NX-600 (*p*_wilcoxon_ = 1.5x10^-5^). The variability across the genome, measured via per base depth of coverage standard deviation was 26.4, 37.8 and 32.2 for the NX-500, NX-600 and TS samples respectively, with the NX-600 SD significantly higher than TS (p_Wilcoxon_ = 0.007). Furthermore, when we considered the number of isolates in which more than 5% of the genome was covered with less than 30x coverage depth, ten isolates (40%) sequenced with NX-500 fell into this category, only one (2%) sequenced with NX-600, and zero for TS. When the depth of coverage threshold was increased to 40x, this number of isolates increased to 21 (84%) for NX-500, 2 (4.3%) for NX-600, and remained zero for TS. At 50x coverage depth, 23 (92%), 8 (17.0%), and only 3 (4.1%) of MTB isolates missed this quality threshold for NX-500, NX-600 and TS respectively (p_exact McNemar_ for the TS vs NX-600 comparison = 0.01) (Fig 3).

### Effect of Isolate Extraction Quality on Sequencing

**Fig 4:**
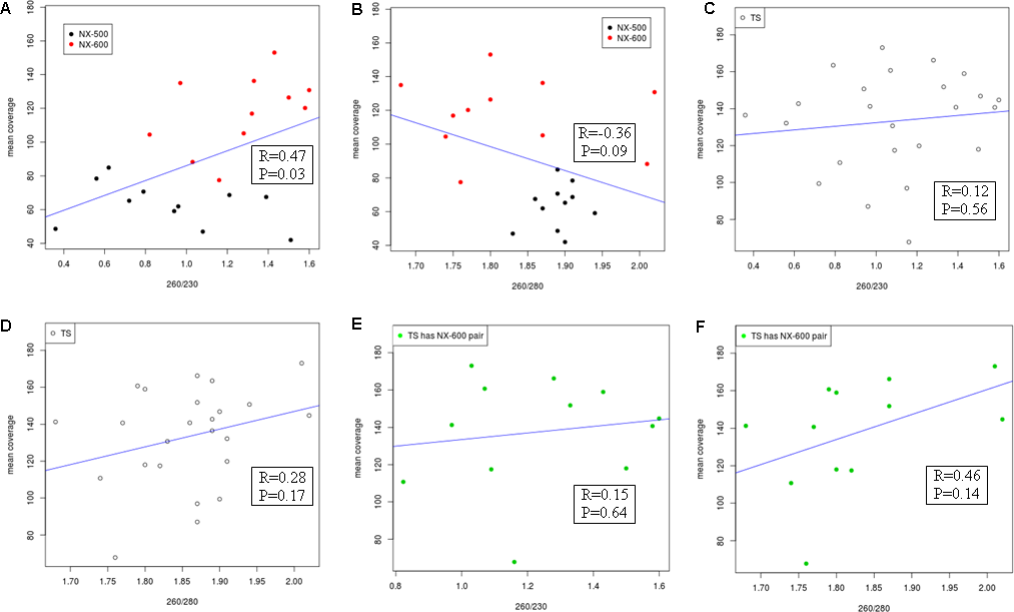
Scatter plot depicting the relationship between A_260/230_ (A, C & E) and A_260/280_ (B, D & F) ratios and mean depth of read coverage. Blue lines illustrate the line of best fit. Isolates deemed outliers, with mean sequencing depth of coverage <25x or >195x, have been excluded. Pearson correlation values and corresponding linear regression *p*-values are expressed.

Input DNA purity was measured spectrophometrically, using A_260/280_ and A_260/230_ ratios [19]. Per base depth of coverage was used as an indicator of output sequencing quality. In order to maximize the statistical power and due to the similar nature of the library preparation protocols, isolate DNA prepared with NX were considered both together and separately as subgroups (NX-500 and NX-600). Twenty-nine samples had WGS data from both NX and TS library preparation methods, and A_260/280_ and A_260/230_ measurements available for analysis. Average A_260/280_ ratios were within expected ranges (mean 1.84), while A_260/230_ ratios were low (mean 1.13), suggesting the presence of a contaminant. We explored the correlation between both ratios and the mean sequencing coverage depth of each isolate. Although there was little evidence for an effect of input DNA quality on sequencing depth of coverage in the TS data, the NX-prepared isolates showed evidence of a positive correlation between A_260/230_ and sequencing coverage depth (*r* = 0.47, *p*-value = 0.03) and a negative association with increasing A_260/280_ values (*r* = -0.36, *p*-value = 0.09) following exclusion of outliers (Fig 4; S2 Fig). In comparing each of the NX technologies separately, it was difficult to assess significance of correlations given the limited isolate numbers; however, a trend was observed towards increased overall depth of read coverage in isolates with higher A_260/230_ ratio values (indicative of cleaner input DNA) in the NX-600 (*r* = 0.45, p-value = 0.16), but not in the NX-500. No correlation was observed in either of these methods when we examined the A_260/280_ ratios.

We then set out to determine whether we might further improve the performance of the NX-600 method by performing an additional DNA clean-up step prior to library construction on an exploratory pair of samples. A_260/280_ ratios for two isolates selected for this analysis were within normal ranges and were approximately equal for isolates pre- and post- column clean-up procedures. However, A_260/230_ ratios were both low, decreasing further as a result of the post-extraction clean-up procedure undertaken. In keeping with the notion that the contaminant may be alcohol-insoluble cell wall constituent carried over, these A_260/230_ values remained low even following an additional ethanol removal step, indicating that some sort of atypical contaminant is present in these (and possibly many of our MTB) genomic templates. Isolates sequenced using TS generated high quality data in both samples regardless of whether additional clean-up was performed. Conversely, when sequenced with NX-600, one isolate generated sufficient sequence quality only with additional post-clean-up processing; the other MTB isolate generated equivalent sequence quality regardless of clean-up (S3 Fig).

### Sequence-specific effects on WGS data quality

ULC regions in both NX (combined NX-500 and NX-600) and TS library preparations were assessed for differences in the frequency of sequence signature occurrence between the technologies on the set of 48 isolates initially sequenced in this experiment. In total, 195 genomic segments (corresponding to 37,383 base pairs (BP), in 82 CDSs) were classified as ULC hotspots in the cohort of isolates sequenced using the NX library method, more commonly than for the those obtained for the TS method (*p*_corr_<0.05). In TS library isolates, 124 regions (corresponding to 32,911 bp in 44 CDSs) were classified as ULC hotspots more commonly than NX (*p*_corr_<0.05) (S2 Table). At 77% GC within the ULC regions missed by NX, and 80% GC in the ULC regions missed by TS (*p*_ttest_ = 0.008), the GC-content in the ULC regions was higher than the background genomic average (roughly 65% for MTB overall), supporting a sequencing bias towards more moderate regions of GC content.

In the TS-prepared samples, ULC regions were located almost exclusively in known repeat regions throughout the MTB chromosome including the PPE and PE-PGRS genes. Gaps in the NX samples occurred in both known repeat regions, as well as additional genes such as *EsxS* and *Esp1* (see S2 Table for complete list). Interestingly, several loci had different segments that were missed significantly more frequently by each of the library methodologies. PE-PGRS2, for example, encoded by a gene spanning 1464-bp, had several small gaps missed by 65% of TS samples, and one large region that was missed by NX in greater than 54% of isolates. Two additional large portions of the PE-PGRS2 CDS proved difficult to sequence in general, and were missed by an additional subset (>20%) of isolates prepared using both methodologies. Additional commonly missed loci encompassing regions that were missed by both NX and TS included genes encoding prophage-like elements (PhiRv1) and additional PE-PGRS and PPE, ESAT-6 regions, among others. Further study will be required to determine whether these regions are in fact absent from the study strains, or whether their absence is the result of inability to accurately map our sequences to these regions.

GLAM2 was used to identify DNA signatures among ULC sequences that were detected significantly more or less commonly among the NX and TS groups. When we examined whether these sequence motifs were detected at varied frequency in genomic regions with ULC compared to regions of higher depth of coverage (>20x sequencing depth of coverage at each position), only a single motif was significantly enriched in the ULC regions. This GC-rich motif CGSCNGSCGKYGCCGSCGSYG (*p*_*corr*_ = 0.05), was poorly sequenced in the isolates prepared using NX. Although this motif was not among the top hits identified by GLAM2 in the TS sequenced isolates, upon further investigation it also was found to be relatively frequently observed among regions missed by the TS library methodology. This novel motif may in fact represent a MTB sequence that is recalcitrant to sequencing via both library methodologies. At this level of significance, there were no additional motifs that were more commonly missed in the ULC regions of TS-prepared isolates or that were commonly missed by both methodologies.

**Fig 5:**
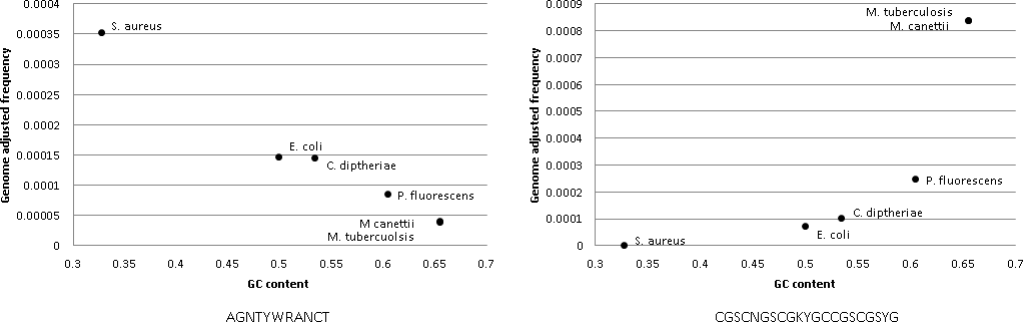
GC content vs. genome size adjusted motif frequency. A) GC-rich motif (CGSCNGSCGKYGCCGSCGSYG) identified that is commonly found in regions of ultra-low sequencing depth of coverage in our MTB isolate analysis. B) AGNTYWRANCT Motif described by Adey *et al (*[23]).

In order to determine whether such motifs are *Mycobacterium*-specific, or have the potential to influence the quality of data generated when investigating other microorganisms as well, the occurrence of our newly identified motif (CGSCNGSCGKYGCCGSCGSYG), as well as the previously described DNA motif from Adey *et al* [23], was measured across the genomes of several organisms. The Adey et al motif was detected less commonly in the MTB H37Rv reference genome than in the other genomes measured (*Staphylococcus aureus*, *Escherichia coli*, *Pseudomonas fluorescens*, *Corynebacterium diphtheriae*, *Mycobacterium canettii*) (Fig 5B). Alternatively, our GC-rich motif identified herein, appeared to occur almost exclusively in MTB and *M*. *canettii* genomes rather than other genomes under investigation. Unsurprisingly, among the tested organisms, it appears that the detection of this GC-rich motif is logarithmically related to the overall GC-content of the organism, increasing dramatically in organisms with higher GC-content, and therefore occurring much more frequently in MTB-specific sequences (Fig 5A).

## Discussion

The importance of adequate depth of sequencing coverage in bacterial WGS analysis cannot be understated. In order to confidently identify SNVs, repetitive sequencing of loci throughout the genome is required in order to ensure that detected genetic variants are not the result of sequencing errors. Indeed, previous work has suggested that to prevent false variant calls, accurate SNV calling in bacterial isolates requires a minimum depth of sequence read coverage of 30-100x in Illumina experiments [24][25][26]. In the context of WGS studies of MTB, a wide range of coverage thresholds have been used to identify SNVs, with few reaching suggested levels of sequencing coverage depth [27][28][5,29]. Findings from previous studies suggest that uniform sequencing depth of coverage may be more difficult to achieve in certain microorganisms owing to both specific features of bacterial physiology and genomic content. In order to mitigate such effects, it is important to ensure that organism-appropriate isolate preparation, from DNA extraction through library preparation and sequencing is used. We herein investigate whether differences in wet lab experimental protocols may improve the quality of data generated when sequencing the MTB genome. Novel findings from this study include the observation of a reduction in depth of sequencing coverage across the MTB genome with use of the NX library preparation protocol, and among samples in which the shorter read generating 500-cycle reagent kit was used. The latter finding is unsurprising as the decreased cycle number corresponds to a reduction in the size of sequence reads, and most likely a reduced ability to assemble sequences either *de novo* or to unambiguously map to a reference [30]. We have investigated whether specific features of the MTB genome and sample preparation quality, negatively impact the data generated via either of these methods.

Previous work has demonstrated that genome content may be partially responsible for variability in the sequencing depth of coverage of bacterial genomes. Studies of the AT- and repeat-rich *Plasmodium falciparum* for example, have demonstrated inconsistent sequencing depth of coverage across the genome [31]. More generally, several studies have illustrated that Illumina SBS is subject to sequencing coverage bias in areas of extremely high and extremely low GC-content, in both whole genome analyses and metagenomic community based analyses [32][33][34][35], with evidence illustrating that this bias may be introduced through the PCR amplification step of the library preparation [7] or during cluster amplification via bridge PCR on the Illumina flowcell[36]. We hereby suggest that the *method* of library preparation is also of high importance in certain organisms. Our data supports this, demonstrating clearly that while regions of high GC-content make up a large proportion of the low coverage genomic regions, that TS prepared isolates may be able to tolerate a slightly higher GC-content burden. The precise mechanism for this improvement is unknown, however, we posit that the higher amount of input DNA required by the TS protocol provides a larger pool of template that is more robust to contaminants, and accounts for the more robust sequencing of organisms with extreme GC-content range. Additionally, the random genomic shearing used by the TS method abrogates the need for enzymatic cleavage based on specific site recognition. Alternative approaches to further enhance WGS quality in the context of GC-rich or AT-rich organisms that were not explored in this investigation, may include optimizing PCR protocol conditions or the use of PCR-free approaches, both of which have been shown to reduce amplification biases [7][34][37].

Additional sequencing difficulty may be attributed to the bioinformatics challenge of mapping reads covering repetitive elements. This difficulty is not specific to MTB, but rather compounds additional challenges that seriously impact the quality of MTB sequencing. MTB loci including PE/PPE-PGRS genes and ESAT-6 [29][38][39] are well described gene families, and are notoriously difficult to analyze using short read sequencing technologies [40], owing to both their GC-richness and their repetition throughout the genome. In this study, many such regions were detected as ULC regions regardless of the method of NGS library preparation. Several of the poorly covered genes, including PE-PGRSs for example, are much longer than the maximal read length of Illumina MiSeq sequencing, thus no single or paired-end read is capable of spanning the entire region of ambiguity. To reduce the ambiguity of mapping in repetitive regions, most mapping approaches involve masking of repetitive regions to prevent erroneous assemblies. These approaches ensure that variant calls are based on sound evidence, at the expense of the removal of data which may or may not contain useful information. For our analysis, masking was not performed, and sequences which mapped equally well to multiple locations were randomly assigned to either of the locations as per the default parameters in SMALT. This procedure, while not typically used for whole genome analysis and phylogeny construction, allowed us to identify regions that achieved low levels of coverage due to inadequate sequencing regardless of the underlying genomic content at these specific regions. Identification of such regions may provide useful additional loci for exclusion in future phylogenetic analyses.

It is also important to note that in order to fully resolve repetitive regions, it is essential to both ensure that high quality data is produced, and to maximize sequence read lengths. In future studies, it will be advantageous to make use of other longer read technologies that are capable of spanning repetitive regions in order to fully resolve ambiguously mapping reads [41]. The use of these long read technologies and the subsequent necessity for appropriate integration of data are, however, outside the scope of this paper and have been well described previously [42][43].

Loci that were poorly sequenced in MTB included regions that contained a CGSCNGSCGKYGCCGSCGSYG motif. Unsurprisingly, this GC-rich motif occurs more frequently in high GC-content organisms, and is detected particularly often in MTB and other related *Mycobacterium* species. Conversely, the previously described Adey *et al*. motif AGNTYWRANCT [23] was detected less commonly among MTB and related mycobacteria. This latter sequence is described as “weakly resembling the reported insertion preference of the native *Tn*5 transposase”[23], and thus genomes containing many copies of this motif may be more readily sequenced than organisms in which it occurs only rarely, although the degree of bias conferred by this sequence is reportedly quite low. None-the-less, the rarer occurrence of this Adey motif in MTB as compared to other organisms, may play a role in the decreased sequencing coverage of MTB, when using the NX platform owing to library preparation bias.

DNA purity was also found to have a substantial effect on data, with the TS protocol more consistently able to provide robust data regardless of the quality of input DNA extract. The cell envelope of MTB is composed of a plasma membrane and a thick outer layer, constructed primarily of complex lipids including mycolic acid[44]. Mycolic acid is a chemically stable, alcohol-insoluble, high molecular weight hydroxy acid [45]. The waxy nature of this component complicates DNA extraction procedures, often leading to contamination of MTB lysates with residual mycolic acid constituents. When measured spectrophotometrically, almost all of our genomic DNA samples showed evidence of some form of contamination comprised of either residual cell components or reagents carried over from the extraction protocol. When we explored the effect of isolate DNA purity on the consistency of sequencing depth of coverage across the genome, we observed a pronounced negative effect upon templates prepared using the NX library method. Our tests investigating the quality of sequencing following use of a commercial template clean-up protocol, while comprising only a pair of samples, further suggests the importance of highly pure template in sequencing mycobacteria and other mycolic acid-containing taxa when using the MiSeq protocol. Enzymatic inhibitors contaminating such samples more broadly affect isolates generated using the NX protocol, an observation that likely results from enzymatic inhibition of the transposase required for efficient DNA fragmentation in the NX procedure. Given that TS relies on mechanical shearing for DNA fragmentation and not enzymatic activity, it is less susceptible to contaminants than is the NX method, as was confirmed by our data. The primary aim of this paper was not to optimize and develop a method for improving upon the purity of DNA yielded from Mycobacterial culture, but rather to provide evidence of alternative library preparation protocols that could mitigate the effects of poor sample quality. For a more detailed discussion of extraction and DNA clean-up protocols, please see [46,47].

Given that this study was conducted on a single microbial species (MTB), it is difficult to predict whether additional microbes might achieve similar improvement in sequence data quality with the adoption of the TS methodology. However, in organisms with similarly complex cell physiology and extremes of GC-content, it may be beneficial to apply the TS library preparation method despite the increased processing time and higher requisite amount of starting template. Thus, we recommend conducting similar critical examination of experimental sequencing data before adopting a specific methodology for large-scale NGS of novel or challenging organisms.

In this study, the relative accuracy and quality of data for several commonly used NGS library methodologies were compared. This work demonstrates that WGS data quality is predicated upon the purity of input DNA template, the composition of the genome in question, and the library preparation methodology used prior to Illumina sequencing. This detailed analysis is the first of its kind to highlight the importance of considering the genomic and physiological nature of the microorganism in question alongside the quality of isolate DNA template produced, in selecting an appropriate experimental method or template workup. We contend that the selection of an appropriate library preparation protocol is necessary in many microbes, including MTB and other Mycobacterium species, with genomes that are at the extremes of GC-content or which have cell membrane properties that influence downstream enzymatic activity.

## Supporting Information

S1 Fig Average PHRED quality scores across forward and reverse reads calculated for the isolates run on the specified technologies. PHRED quality scores which are <20 are flagged (red). Mean score depicted by the blue line included in the image.
(TIF)

S2 Fig Scatter plot depicting the relationship between A_260/230_ and A_260/280_ ratios and mean depth of read coverage. Blue lines illustrate the line of best fit for all points, with black and red lines representing lines of best fit for corresponding groups. Isolates deemed outliers, with mean sequencing depth of coverage <25x or >195x, have been excluded. Pearson correlation values and corresponding linear regression *p*-values are expressed
(TIF)

S3 Fig GView image of the pangenome created for one of the isolates that underwent MiSeq sequencing of both raw extraction and cleaned-up DNA preparation. Areas of the pangenome that are not coloured for a specific preparation have been filtered out due to low sequencing depth of coverage (<20x).
(TIF)

S1 Table Basic statistics for each of the study groups included in this analysis. Described means of sample standard deviation, median and interquartile range (IQR) are calculated based on the genome-wide depth of coverage in each sample, at each locus. ^†^ represents significantly different values.
(DOCX)

S2 Table List of loci designated as ultra-low coverage (<5x coverage of region extending at least 10 base pairs long). (XLSX)
