# Supplementary figures and images for: Comparison of Sample Preparation Methods Used for the Next-Generation Sequencing of Mycobacterium tuberculosis
Source: PLoS One. 2016 Feb 5;11(2):e0148676. doi: 10.1371/journal.pone.0148676 (PMC4744016; doi:10.1371/journal.pone.0148676)

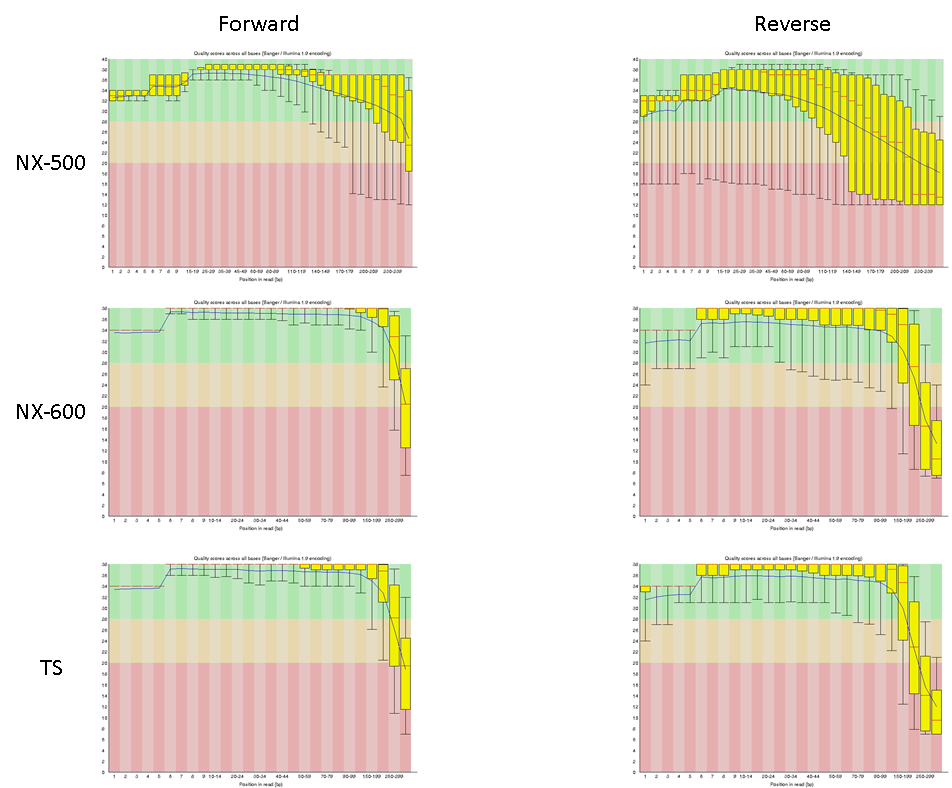

Supplement: S1 Fig — PHRED quality scores which are <20 are flagged (red). Mean score depicted by the blue line included in the image. (TIF) [file pone.0148676.s001.tif]

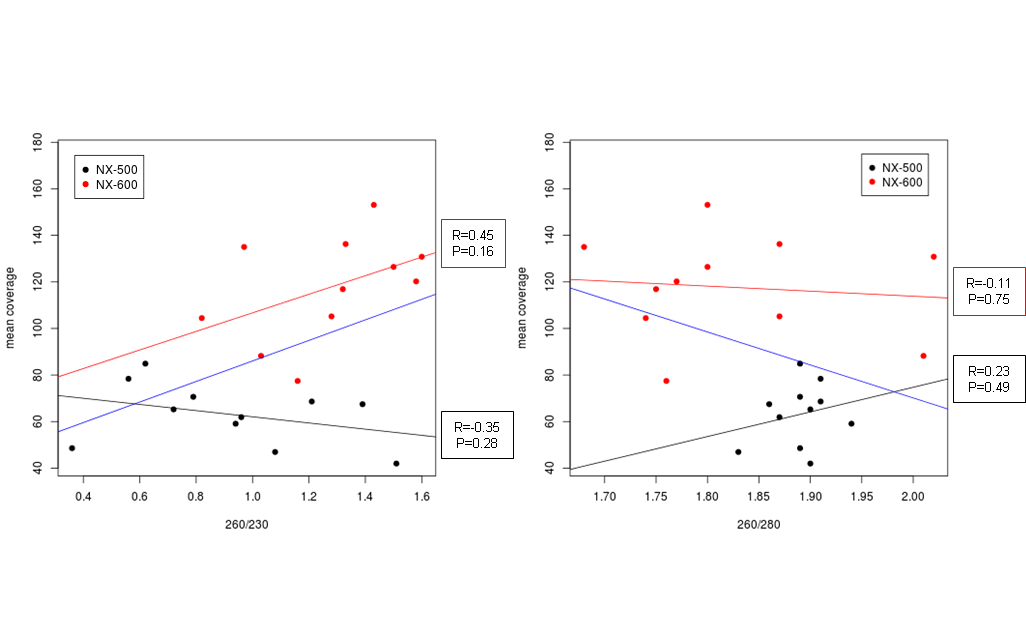

Supplement: S2 Fig — Blue lines illustrate the line of best fit for all points, with black and red lines representing lines of best fit for corresponding groups. Isolates deemed outliers, with mean sequencing depth of coverage <25x or >195x, have been excluded. Pearson correlation values and corresponding linear regression p-values are expressed (TIF) [file pone.0148676.s002.tif]

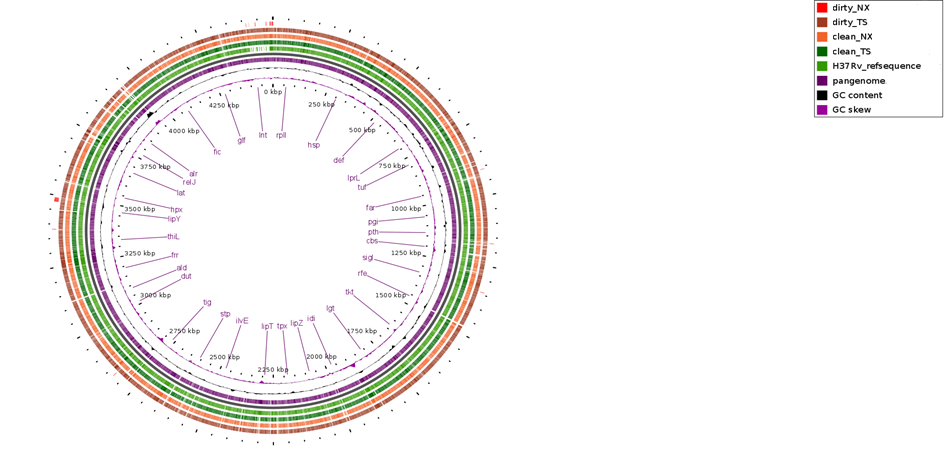

Supplement: S3 Fig — Areas of the pangenome that are not coloured for a specific preparation have been filtered out due to low sequencing depth of coverage (<20x). (TIF) [file pone.0148676.s003.tif]

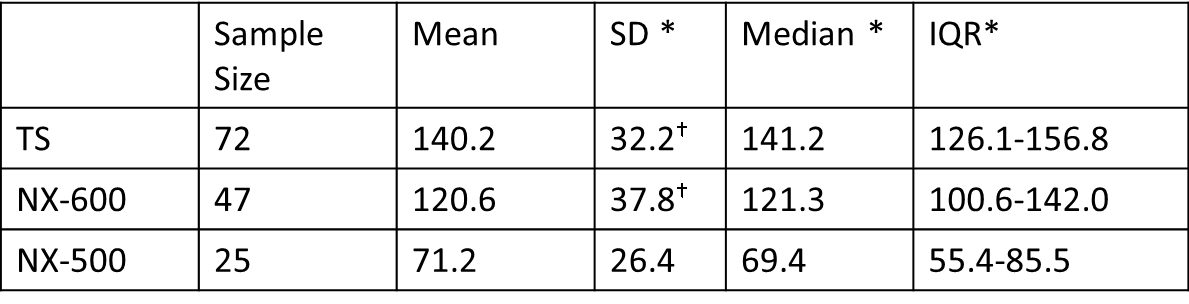


* Mean of median and IQR included on this table.

† represents significantly different values

Supplement: S1 Table — Described means of sample standard deviation, median and interquartile range (IQR) are calculated based on the genome-wide depth of coverage in each sample, at each locus. † represents significantly different values. (DOCX) [file pone.0148676.s004.docx]
